# Comparison of Disease Activity in SPMS and PPMS in the Context of Multicenter Clinical Trials

**DOI:** 10.1371/journal.pone.0045409

**Published:** 2012-10-01

**Authors:** Rotem Orbach, Zhenming Zhao, Yong-Cheng Wang, Gilmore O’Neill, Diego Cadavid

**Affiliations:** 1 MS Clinical Development Group, Biogen Idec, Cambridge, Massachusetts, United States of America; 2 Biostatistics, Biogen Idec, Cambridge, Massachusetts, United States of America; 3 Multiple Sclerosis Center at Sheba Medical Center, Tel-Hashomer, Israel; Charité University Medicine Berlin, Germany

## Abstract

**Background:**

Retrospective single center natural history studies have shown that times to reach disability milestones and ages at which they are reached are similar in primary (PPMS) and secondary (SPMS) progressive multiple sclerosis suggesting that they may be phenotypic variations of the same disease.

**Objective:**

Here we compared longitudinal disease activity in SPMS and PPMS in the context of international multicenter clinical trials.

**Methods:**

We analyzed all objective outcome measures that were systematically collected over 2 years for all subjects randomized to placebo arms in one SPMS and one PPMS clinical trial over the last decade. Conventional and exploratory definitions of clinical disease activity were used. Disease activity was analyzed in 3 different categories intermittent activity, progression, and improvement. Conventional MRI measures and one patient reported outcome measure of quality of life were included when available for comparison. Heat maps were drawn for all results followed by hierarchical clustering.

**Results:**

There were 101 outcome variables from 206 SPMS subjects and 79 outcome variables from 135 PPMS subjects. The comparison revealed that SPMS and PPMS subjects exhibited similar disease activity over 2 years in all but two of the variables in common worsening in the EDSS sensory system was more common in PPMS while worsening on the 9 hole PEG was more common in SPMS. Intermittent activity was the most common pattern of disease activity in SPMS and PPMS. Clinical worsening and improvement occurred at similar frequency in both.

**Conclusion:**

Longitudinal disease activity was nearly identical in SPMS and PPMS subjects in the context of the two multicenter international clinical trials we examined.

## Introduction

Although multiple sclerosis (MS) usually begins with a relapsing-remitting course in 85% of patients, over long term follow up the majority of patients develop sustained accumulation of disability referred to as secondary progressive MS (SPMS). About 15% of MS patients develop sustained accumulation of disability from clinical onset without reporting a preceding period of clinical relapses and remissions and are referred to as primary progressive MS (PPMS) [1]. Although some disability is acquired via incomplete recovery from relapses, it is the sustained loss of neurological function characteristic of the progressive forms of MS that is responsible for most of the disability that accumulates in MS patients [2]. There is an unmet need for better outcome measures of disease progression in SPMS and PPMS. There is also an important need to better understand the similarities and differences between SPMS and PPMS. Most previous studies on this subject have been based on large single center cohorts [3], [4], [5]. One such study revealed that the time to reach disability milestones and ages at which these landmarks are reached in the progressive forms of MS follow a predefined schedule not obviously influenced by relapses [6], [7]. Based on this it has been proposed that PPMS and SPMS might be regarded as essentially similar [6]. However, the current regulatory guidance views PPMS and SPMS as different diseases.

Here we studied for the first time differences in disease activity between SPMS and PPMS in the context of multicenter studies. For this we examined all available objective clinical outcome measures that were systematically collected at quarterly scheduled visits over 2 years in subjects randomized to placebo in the IMPACT SPMS [8] and the OLYMPUS PPMS [9] clinical trials. Three different patterns of clinical activity changes were studied: intermittent, progression, and improvement. We then applied the concept of “heat maps” from biological gene expression analysis to compare disease activity in PPMS and SPMS across selected variables in common. When analyzing multidimensional, quantitative datasets, the comparison of two or more groups is a common task [10]. Typical sources of such datasets are often experiments in biology, physics or engineering but less often clinical research. One common way to analyze complex datasets is to filter it using statistical methods and then run clustering algorithms to group similar variables. The clustering results can be visualized using heat maps, which show differences between groups as changes in color. We applied this approach to the placebo arms of two 2-year clinical trial datasets of SPMS and PPMS subjects. The results revealed that for most outcome measures examined PPMS and SPMS exhibited remarkably similar disease activity.

## Methods

### Study Subjects

All data were from the placebo arms of two large randomized, prospective, double-blinded multicenter clinical trials of progressive MS, IMPACT and OLYMPUS. IMPACT was a 2-year study in North America, Europe, and Israel that evaluated the efficacy of 60 mcg weekly injections of intramuscular interferon beta-1a (IFNb-1a, AVONEX®) vs. placebo for the treatment of SPMS [8]. OLYMPUS was a phase II/III trial from 60 centers in the US and Canada in which subjects were randomly assigned 2:1 to receive the B cell depleting monoclonal antibody rituximab (two 1,000 mg intravenous infusions every 24 weeks through 96 weeks) or placebo [9]. The study populations and primary and key secondary outcomes of these trials were previously published [8], [9]. Informed consent was obtained for all subjects enrolled in these trials.

### Measures of Disease Activity

We examined all clinical efficacy outcome measures that were systematically collected at the scheduled visits every 3 months for 2 years. These included measures of clinical disability like the EDSS [11] and measures of physical and cognitive function like the multiple sclerosis functional composite (MSFC) [12]. Conventional MRI measures from yearly brain MRI scans were also examined, including the number of new and/or enlarging brain MRI lesions and the change in brain volume from baseline using either brain parenchymal fraction (in IMPACT) or change in whole brain volume (in OLYMPUS). We also examined one patient reported outcome measure instrument, the multiple sclerosis quality of life inventory (MSQLI) [13], that had been collected only from English speaking subjects at yearly intervals in the IMPACT study. Altogether we analyzed disease activity over 2 years using 101 outcome measures in IMPACT placebo subjects and 79 in OLYMPUS placebo subjects. Table 1 lists the operational definitions for all clinical and MRI measures of disease activity divided in 3 categories: intermittent activity, progression, and improvement.

**Table 1:** Definitions of disease activity used in the analysis.

MRI
*Intermittent*“yes” if there is any new or enlarging T2/FLAIR lesion or gadolinium lesion at any time including baseline, otherwise “no”
*Progression 1*“yes” if brain parenchymal fraction measurement at 24 months is lower than baseline by at least 1 SD, otherwise “no”
*Progression 2*“yes” if brain parenchymal fraction measurement at 24 months is lower than baseline by at least 0.5 SD, otherwise “no”
*Progression 3*“yes” if brain parenchymal fraction measurement at 24 months is lower than baseline by at least 0.25 SD, otherwise “no”
**EDSS Total Score**
*Intermittent*“yes” if there is any increase in score comparing to the prior measurement of at least X point followed by any decrease in next 6 months (either after 3 M or 6 M) (X = 1 if baseline EDSS < = 5.5; X = 0.5 if baseline EDSS > = 6), otherwise “no”
*Progression 1*“yes” if there is any increase in score comparing to the baseline of at least X point and there is no decrease after that (can stay stable or increase further) (X = 1 if baseline EDSS < = 5.5; X = 0.5 if baseline EDSS > = 6), otherwise “no”
*Progression 2*“yes” if (score of any time point - score at baseline) are positive for X number of times (X > = 5, 6, 7, or 8 out of 8), otherwise “no”
*Improvement*“yes” if both scores of the last 2 visits are smaller than baseline by at least X point (X = 1 if baseline EDSS < = 5.5; X = 0.5 if baseline EDSS > = 6), otherwise “no”
**EDSS Sub-System Scores**
*Intermittent*“yes” if there is any increase in score of at least 2 point compared to prior measurement followed by any decrease in next 6 months (either after 3 M or 6 M), otherwise “no”
*Progression 1*“yes” if there is any increase in score comparing to the baseline of at least 1 point and there is no decrease after that (can stay stable or increase), otherwise “no”
*Progression 2*“yes” if (score of any time point - score at baseline) are positive for X times (X > = 5, 6, 7, or 8 out of 8 visits), otherwise “no”
*Improvement*“yes” if both scores of the last 2 visits are smaller than baseline by at least 1 point, otherwise “no”
**MSFC components (note: PASAT has different direction)**
*Intermittent*“yes” if there is any increase in time compared to the prior measurement of at least 20% followed by any decrease over the following 6 months (either after 3 M or 6 M), otherwise “no”
*Progression 1*“yes” if there is any increase in time compared to the baseline of least 20% and there is no decrease after that (can stay stable or increase), otherwise “no”
*Progression 2*“yes” if (time of any time point - time at baseline) are positive for X number of times (X > = 5, 6, 7, or 8 out of 8 visits), otherwise “no”
*Improvement*“yes” if both times of the last two study visits are smaller than baseline by at least 20%, otherwise “no”
**MSQLI**
*Progression*“yes” if at least two scores at (month 15 and month 24) are lower than baseline, otherwise “no”
*Improvement*“yes” if at least two scores at (month 15 and month 24) are higher than baseline, otherwise “no”

### Statistical Analysis

To measure disease activity, 101 variables in the IMPACT study and 79 in the OLYMPUS study were derived by using the original raw data according to pre-specified criteria (Table 1). There were 2 definitions of clinical progression for the EDSS and MSFC components, definition 1 based on thresholds and definition 2 based on consistency of change. MRI progression was measured based on thresholds of change from baseline over 2 years. Intermittent activity and progression were coded to 1/0, and improvement was coded to −1/0. Histograms were made to assess the sensitivity of the proposed definitions and missing data. A sensitivity analysis was performed to evaluate the definition 2 of progression by using the definition 1 of the EDSS total score as anchor. This revealed that a progression 2 definition that used worsening in at least 6 out of 8 visits (by any value) from baseline over 2 years captured progression with similar sensitivity and specificity as the traditional definition of progression using the total EDSS score change. Accordingly, the threshold of ≥6/8 was selected for definition 2 of clinical progression (Table 1). Hierarchical clustering of the individual subjects was performed for selected variables based on the Euclidean dissimilarity and average linkage and displayed by heat maps. Fisher’s exact test was used to test the difference of the outcome measures between SPMS and PPMS and the difference between progression and improvement in MSQLI components. The Benjamini-Hochberg method (FDR) was used to adjust for multiple comparisons. Data were analyzed using the SAS 9.1 software package (SAS Institute, Cary NC) and R 2.10 (The R Foundation for Statistical computing).

## Results

### Baseline Characteristics

The demographic and clinical baseline characteristics of the placebo arms of the IMPACT and OLYMPUS trials were relatively similar except for the expected history of previous relapses that was present only in SPMS (Table 2). There were more females in IMPACT (64%) than OLYMPUS (55%). Gadolinium enhancement at baseline was also more common in IMPACT (34.4%) than OLYMPUS (25.2%). MS duration was also longer for IMPACT (average 16.7 years) than OLYMPUS (average 9 years). We did not have information on the time since conversion from RRMS to SPMS in IMPACT. The PASAT scores were nearly identical while the EDSS, T25FW, and 9HP scores were slightly higher in IMPACT than OLYMPUS. There was a much higher previous use of interferon beta in the OLYMPUS study because the IMPACT study design excluded subjects with any previous therapy with any interferon beta product.

**Table 2:** Baseline characteristics of the SPMS and PPMS subjects included in the analysis.

	IMPACT (n = 219)	OLYMPUS (n = 147)
Characteristic		
Age	47.9 (7.7) a	49.6 (8.7)
Gender, Female, n (%)	141 (64)	81 (55)
Number of relapses in past year	0.38 (0.49)	N/A
Gd-enhancing lesions, count >0, n (%)	75 (34.4)	37 (25.2)
Disease Duration (years)		
Since first symptom	16.7 (9.0)	9.0 (6.8)
Since diagnosis	10.5 (7.5)	3.8 (4.2)
Prior treatment with INF-β or GA, n (%)		
Ended >90 days before trial entry	2 (1)^b^	45 (30.6)
Ended ≤90 days before trial entry	0	6 (4.1)
EDSS score	5.2 (1.1)	4.7 (1.4)
MSFC components		
T25FW (sec)	14.6 (15.4)	11.6 (15.8)
9HPT (sec)	33.2 (30.0)	29.9 (13.0)
PASAT-3 (sec)	46.8 (12.3)	46.7 (12.6)

a All of the values are mean (SD), except Gender and Prior MS treatment, and Gd-enhancing lesions.

b These two subjects with prior MS treatments were on GA, which ended >180 days before trial entry.

### Disease Activity in SPMS

**Figure 1:**
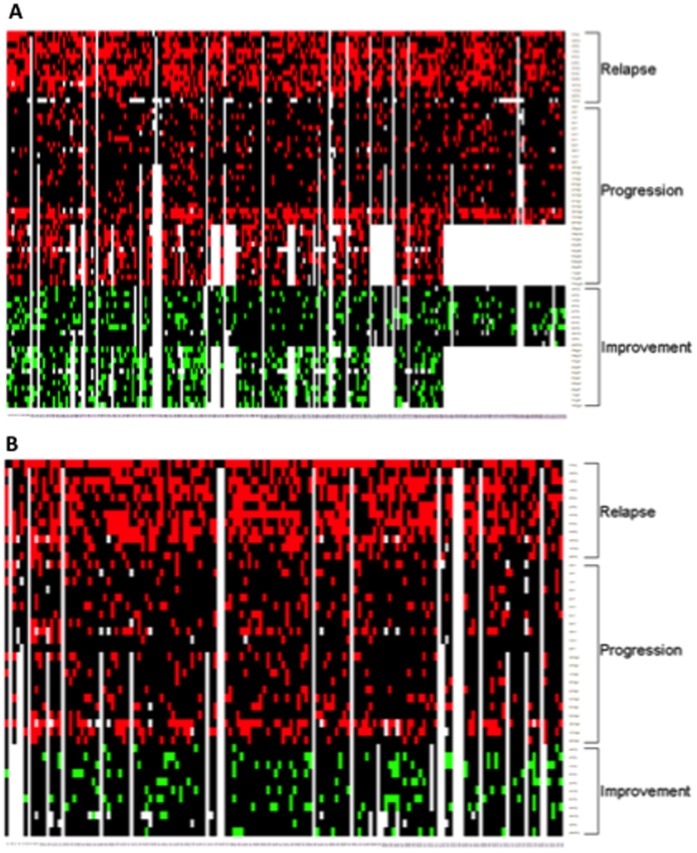
Heat map display of disease activity at the individual level. The heat maps have patients on columns, and outcome measures on rows. Red indicates intermittent activity or progression, green indicates improvement, black indicates no change, and white indicates missing value. The data are from 2-year studies in SPMS (panel A) and PPMS (panel B) subjects randomized to placebo in the IMPACT [8] and OLYMPUS [9] multicenter clinical trials. All calculations were performed based on the definitions listed in Table 1. Notice that progression was most often detected using definition 2 with the T25FW and the 9HP.

First we investigated disease activity in SPMS. We did this in 219 subjects randomized to placebo in the IMPACT study and followed for 2 years examining 101 different definitions of disease activity.The analysis revealed that the median percentage of SPMS subjects experiencing intermittent activity over 2 years was ∼22% (Table 3). Intermittent MRI activity (based on new or enlarging T2 brain MRI lesions) was by far the most common finding of disease activity at 64.6%, while intermittent clinical activity on the EDSS pyramidal system was the least common experienced by <4% of the subjects. The mean number of new T2 lesions after 2 years was 2.61. The median percentage of subjects experiencing clinical progression based on definitions 1 was 11.8% (Table 3). The total EDSS score and the T25FW were the most sensitive measures to capture clinical progression using definition 1 at just below 20%. The PASAT was the least sensitive at only 1.5%. The median percentage of subjects experiencing clinical disease progression across all definitions 2 was 12% (Table 3). The definition 2 of clinical progression using the MSFC components were the most responsive at detecting disease progression over 2 years at ∼58% with the T25FW and the 9HP and ∼23% with the PASAT. The median percentage of subjects experiencing clinical improvement was 13.6%, with the EDSS bowel and bladder and sensory systems being the most frequently improved and the 9HP test the least frequently improved (Table 3). The analysis of the MSQLI components for the English speaking SPMS subjects showed that progression was most frequently reported in mental status, improvement was most frequently reported in fatigue, and the visual system was the least changed of the patient reported measures (Table 4). The median percentage of subjects experiencing progression across MSQLI components (35%) was higher than the median percentage experiencing improvement (30%). Heat map display of disease activity at the individual level in the placebo arm of IMPACT is shown in Figure 1 (panel A).

**Table 3:** Comparison of disease activity over 2 years in placebo SPMS versus placebo PPMS by various definitions of intermittent activity, progression, and improvement.

	SPMS (%)	PPMS (%)	p-value
**Average of Intermittent Activity (median)** a	21.6	19.9	
MRI	64.6	68.0	0.79^b^
EDSS Total Score	28.2	32.1	0.74
EDSS Cerebral	24.3	14.2	0.21
EDSS Visual	20.9	11.9	0.22
EDSS Brain Stem	19.9	19.4	1
EDSS Pyramidal	3.4	7.5	0.38
EDSS Sensory	14.1	22.4	0.24
EDSS Cerebellar	22.3	19.4	0.79
EDSS Bowel & Bladder	17.5	11.2	0.38
T25FW	50.0	48.4	0.94
9HP	32.4	20.3	0.17
PASAT	20.4	30.6	0.22
**MRI Progression** c			
1 (≥1 SD)	1.9	1.6	1
2 (≥0.5 SD)	6.5	5.5	0.92
3 (≥0.25 SD)	36.8	26.6	0.28
**Average of Clinical Progression 1 (median)**	11.8	13.4	
EDSS Total Score	19.2	22.5	0.75
EDSS Cerebral	7.8	11.4	0.67
EDSS Visual	14.9	9.1	0.38
EDSS Brain Stem	11.9	6.1	0.31
EDSS Pyramidal	11.3	13.4	0.80
EDSS Sensory	6.0	19.5	0.008*
EDSS Cerebellar	15.6	17.2	0.94
EDSS Bowel & Bladder	16.1	14.4	0.94
T25FW	19.8	29.8	0.22
9HP	10.7	6.2	0.42
PASAT	1.5	1.5	1
**Average of Clinical Progression 2 (median)**	12.3	14.4	
EDSS Total Score	19.1	23.2	0.70
EDSS Cerebral	6.7	9.6	0.70
EDSS Visual	12.3	12.0	1
EDSS Brain Stem	11.8	7.2	0.55
EDSS Pyramidal	8.2	11.2	0.74
EDSS Sensory	7.7	19.2	0.04*
EDSS Cerebellar	10.8	19.4	0.22
EDSS Bowel & Bladder	13.3	12.8	1
T25FW	58.5	56.5	0.94
9HP	58.7	40.5	0.04*
PASAT	22.6	14.4	0.29
**Average of Clinical Improvement (median)**	13.6	12.4	
EDSS Total Score	5.3	4.1	0.94
EDSS Cerebral	13.6	16.5	0.75
EDSS Visual	14.7	20.7	0.50
EDSS Brain Stem	16.2	13.2	0.75
EDSS Pyramidal	9.5	12.4	0.74
EDSS Sensory	22.0	9.9	0.07
EDSS Cerebellar	17.5	18.2	0.99
EDSS Bowel & Bladder	22.5	15.7	0.40
T25FW	2.3	4.7	0.65
9HP	0.0	0.9	0.70
PASAT	5.3	10.1	0.42

a For each clinical disease activity pattern (intermittent, clinical progression 1, clinical progression 2, and improvement) we present the median percentage as an average of all the measurements within each category.

b All P values have been adjusted for multiple comparisons.

c Brain parenchymal fraction (BFP) was measured in IMPACT study while whole brain volume was measured in OLYMPUS study. For the purpose of multiple comparison correction, MRI progression was considered as a single variable (with MRI progression 1, 2, and 3 being presented as a sensitivity analysis).

### Disease Activity in PPMS

A similar analysis of disease activity was done for the 147 PPMS subjects randomized to placebo and followed for 2 years in the OLYMPUS study based on the availability of 79 definitions The median percentage of subjects experiencing intermittent activity ∼20% (Table 3), about the same as in SPMS. New or enlarging brain MRI lesions showed the highest percentage of relapsing activity at 68% while transient worsening in the pyramidal EDSS system was the lowest (7.5%; Table 3). The mean number of new T2 brain lesions at month 24 was 2.33. Progression of disability according to definitions 1 averaged 13.4%. The T25FW was the most sensitive measure capturing disease progression using definition 1 at 29.8%. Disease progression based on definition 2 averaged 14.4% (Table 3). T25FW using the definition 2 of progression classified the highest number of PPMS subjects as progressors at 56.5% followed by 40.5% with the 9HP (Table 3). Clinical improvement was observed in 12.4% of subjects. The analysis of improvement showed that the visual EDSS score was the most improved at 20.7%, while the 9HP was the least improved at <1%. There was no information on MSQLI from the OLYMPUS trial. Heat map display of disease activity at the individual level is shown in Figure 1 (panel B).

**Figure 2:**
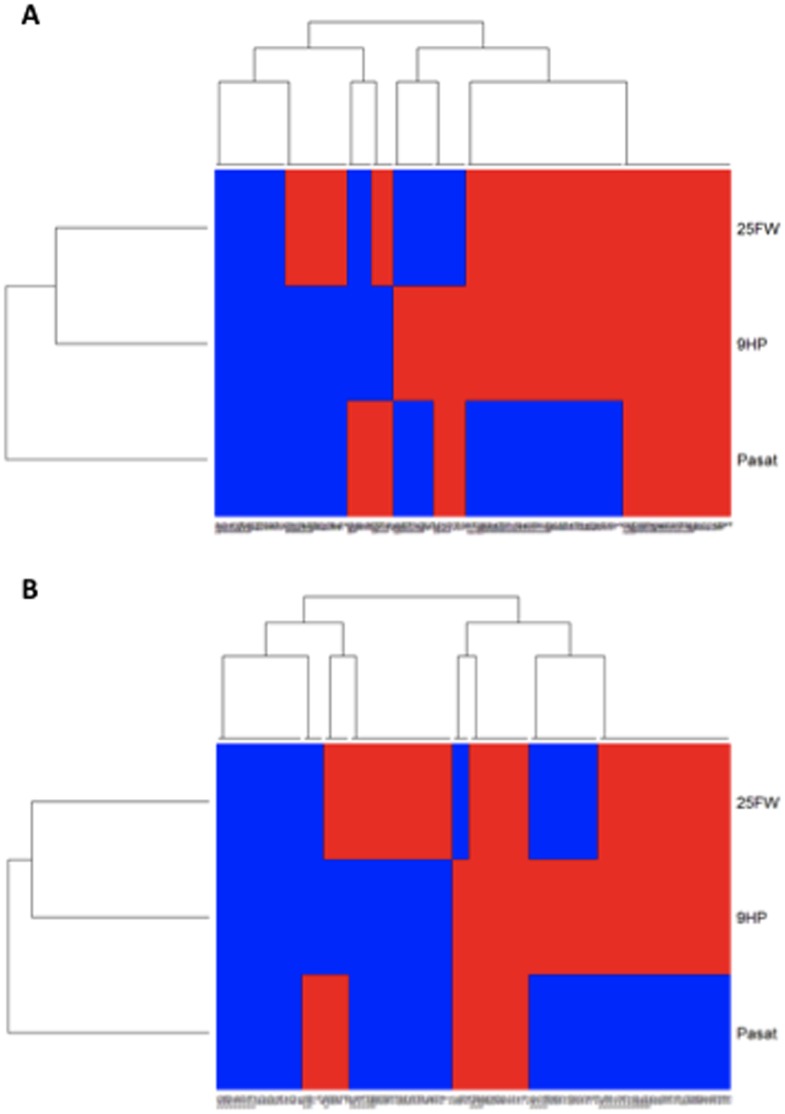
Hierarchical clustering analysis of disease progression using consistent worsening of physical and cognitive function using the multiple sclerosis functional composite. Consistent worsening was determined by using the progression 2 definition (at least 6/8 worse than baseline; Table 1). Study subjects are on columns. Red indicates “progressor”, blue indicates lack of progression. The data are from 2-year studies in SPMS (a) and PPMS (b) subjects randomized to placebo in the IMPACT [8] and OLYMPUS [9] clinical trials.

### Comparison of Disease Activity between SPMS and PPMS

SPMS and PPMS showed very similar disease activity over 2 years. Only 3 out of the 46 outcome measures in common in the two studies showed significant differences and only one was highly significant: In PPMS there was greater progression in the EDSS sensory system by either definition 1 (p = 0.008) or definition 2 (p = 0.04) and in SPMS there was greater progression in the 9HP but only using definition 2 (p = 0.04, Table 3). In both groups the most sensitive measure of disease activity was the development of new brain MRI lesions, observed in 64.6% of SPMS and 68% of PPMS subjects. In both groups disease activity was observed more frequently with an intermittent pattern, affecting ∼20–22% of subjects across all the assessments in both trials (Table 3). There were no significant differences between SPMS and PPMS in the frequency of MRI progression as measured by the loss of brain volume over 2 years regardless of the cut off used (1, 0.5, or 0.25 standard deviation change from baseline; Table 3).

### Cluster Analysis of Disease Activity in SPMS and PPMS

A cluster analysis of disease activity over 2 years at the individual subject level was performed using the definition 2 of clinical progression with the T25FW, 9HP, and PASAT (Figure 2). For this purpose, data from 191 SPMS and 120 PPMS subjects was available. A total of 8 clusters of activity were identified in both SPMS and PPMS subjects: (1) isolated progression in walking; (2) isolated progression in upper extremity function; (3) isolated progression in cognitive function; (4) combined progression in walking and upper extremity function; (5) combined progression in walking and cognitive function; (6) combined progression in upper extremity and cognitive function; (7) progression in all 3 functions (walking, arm function, and cognition); and (8) a cluster of subjects that did not demonstrate progression in any of the functions examined. Among the SPMS subjects, 20.9% demonstrated progression in all 3 domains compared to 11.7% in the PPMS subjects (p = 0.045). Isolated progression in walking occurred more frequently in PPMS (20%) than in SPMS (12%) (p = 0.07). Isolated progression in cognitive function was similarly infrequent in SPMS (4.7%) and PPMS (4.2%). When taking into consideration all possible clusters that included progression in cognition (either isolated or combined with other functions), it was more common in SPMS (36.1%) than in PPMS (24.1%) (p = 0.03). The cluster that did not demonstrate any functional worsening occurred at similar frequency in the two trials, 13.6% in SPMS and 16.7% in PPMS (Figure 2).

**Table 4:** Frequency of patient reported progression and improvement in English speaking subjects with SPMS from the IMPACT study.

	Progression (%)	Improvement (%)	p-value
Average of MSQLI components (median)	34.9	30.0	
SF36 Physical Components	37.3	32.2	0.91
SF36 Mental Components	43.2	25.4	0.04*
Modified Fatigue Impact Scale (MFIS)	36.2	39.4	0.95
MOS Pain Effects Scale (PES)	34.4	31.2	0.95
Sexual Satisfaction Scale (SSS)	29.5	18.2	0.38
Bladder Control Scale (BLCS)	37.6	21.6	0.04*
Bowel Control Scale (BWCS)	29.3	20.3	0.38
Perceived Deficits Questionnaire (PDQ)	34.9	34.9	1
Impact of Visual Impairment Scale (IVIS)	14.8	14.8	1
Mental Health Inventory (MHI)	38.1	31.7	0.78
Modified MOS Social Support Survey (MSSS)	32.5	30.0	0.95

Note: P values have been adjusted for multiple comparisons.

## Discussion

Although SPMS and PPMS are similar in many respects [6], [14], [15], [16], they also exhibit some differences [17], [18]. To further examine the important question of whether SPMS and PPMS may be regarded as essentially similar [6] and to investigate potential novel endpoints for progressive MS trials, we compared the natural history of disease activity in SPMS and PPMS using outcome variables systematically collected over 2 years in two international multicenter clinical trials. The main findings are the following: 1) For most variables examined, SPMS and PPMS subjects exhibited similar disease activity. 2) Intermittent activity was common and occurred with similar frequency in SPMS and PPMS. 3) SPMS and PPMS subjects developed new brain MRI lesions and brain atrophy at similar rate. 4) Clinical progression occurred at similar rate in SPMS and PPMS and was observed nearly twice more often with definition 2 than with definition 1. 5) Progression in the T25FW and the 9HP as measured by definition 2 classified the largest number of SPMS and PPMS subjects as progressors. 6) Improvement occurred at similar frequency as progression in both SPMS and PPMS. 7) Per subject self-report progression occurs slightly more often than improvement.

The frequency of subjects with clinical exacerbations (MS relapses) was much higher (37%) in IMPACT [8] than in OLYMPUS (3.4%) [9]. However, the percentage of subjects with gadolinium enhancement at baseline (Table 2) and treated with corticosteroids was only slightly higher in IMPACT than OLYMPUS (31% in IMPACT versus 24% in OLYMPUS). Our analysis of disease activity over 2 years using both traditional and novel definitions showed that disease activity in SPMS and PPMS occurred at the same rate in nearly all the variables we examined (Table 3). Unexpectedly, intermittent activity occurred at higher frequency than disease progression or improvement both clinically and by MRI. However, the rate of MRI visible new brain lesion development was quite low, averaging about 1 per year. This is consistent with prior studies showing that patients with PPMS have slow rates of new brain lesion formation [19]. One study found that 44% of patients with PPMS demonstrated one or more new brain lesions over a 1-year follow up period [19]. A 2 year longitudinal study of 39 PPMS patients showed that the majority (91%) of the total new T2 lesion volume corresponded to enlargement of pre-existing lesions rather than to formation of new lesions (9%) [20].

It is possible that our clinical definitions of intermittent activity measured examiner and/or subjects’ noise rather than true change. We believe this is unlikely for the following reasons: (1) To minimize false positive findings due to measurement noise we selected robust thresholds, for example ≥2 points increase for the individual EDSS system scores [21] and ≥20% worsening for the MSFC components [22]; changes of these magnitude occur only infrequently due to noise. (2) There was a similar high frequency of new brain MRI lesions in the placebo subjects from the two trials. (3) Lowering the threshold of worsening in the intermittent activity definitions of the EDSS individual system scores from 2 points (Table 1) to 1 point nearly doubles the frequency of intermittent activity (not shown); this points to the greater specificity of the 2 point threshold.

How can one explain the much lower frequency of relapses in PPMS than in SPMS that occurs not only in clinical practice but also in the context of prospective frequent follow up in clinical trials such as OLYMPUS? One possibility is that there are differences in pathophysiology accounting for greater relapsing activity in SPMS. However, our analysis of clinical and MRI measures revealed similar frequency of intermittent disease activity in SPMS and PPMS both clinically and by MRI. An alternative possibility is that there could be under recognition by PPMS subjects and/or their caretakers, relatives, and significant others of symptoms of relapsing activity in the context of sustained disease progression from onset. Another possibility is that patients who end up diagnosed with PPMS are those who underreport intermittent or acute symptoms. This appears unlikely because the detection of MS exacerbations in the OLYMPUS PPMS trial was 9 times lower than in IMPACT despite similar on study monitoring in both trials [9], [23]. Finally, it is also possible that PPMS subjects experience less dynamic changes during relapsing activity than RRMS subjects. A more detailed analysis of symptom profile dynamics might be the tool of choice to clarify this. As the field of MS has not yet agreed upon a sensitive dynamic measure for relapsing activity this should be an area of further research in the future.

The remarkable similarities in disease activity between IMPACT and OLYMPUS may be explained because these trials enrolled highly selected patients that are not representative of the general population of SPMS and PPMS patients in the clinic. We believe this is unlikely as the inclusion/exclusion criteria covered a wide segment of the MS population with a wide age range (18–60 years old in IMPACT and 18–65 years old in OLYMPUS), wide EDSS range (3.5–6.5 for IMPACT and 2.5–6.5 for OLYMPUS), and wide range of disease duration (at least 12 months for both trials) [8], [24]. Furthermore, both trials used precise diagnostic criteria for enrollment.

The traditional tool to measure progression of disability in MS has been the EDSS [11], which is unresponsive to disease progression in the EDSS range characteristic of SPMS and PPMS, between 3.5 and 7 [25], [26]. This was confirmed in our present analysis: the total EDSS score detected sustained progression of disability in only about 20% of the SPMS and PPMS subjects over 2 years. This is problematic for therapeutic clinical trials when the demonstration of drug efficacy depends on the progression of the placebo arm and can result in the need to enroll a large number of subjects who will not progress and therefore will not contribute to answer the efficacy question of the trial. One way to increase the percentage of subjects progressing on EDSS is using a definition of progression based on confirmation on a second examination over shorter term follow up, e.g. 3 months instead of 6 months. However, this is problematic because the 3 month confirmed EDSS measures disability related to intermittent activity [27]. Similar low responsiveness was observed for the definition 1 of progression for all 3 MSFC components (Table 3). In contrast, the definition 2 of progression applied to the T25FW and the 9HP classified 2 to 3 times more SPMS and PPMS subjects as progressors than the EDSS total score (Table 3). This finding is consistent with our previous observation that the physical functional components of the MSFC are more responsive to change than the EDSS in subjects with progressive MS [28]. As expected for a progressive MS population, we found very low frequency of confirmed improvement at the end of the 2 years in T25FW and 9HP in both SPMS and PPMS (<5%, Table 3).

The finding that the physical components of the MSFC using the definition 2 are sensitive assessors of progression over 2 years allowed us to compare the patterns of disease progression at the individual level in SPMS and PPMS using clustering algorithms and heat map display [10]. This analysis revealed that the consistent loss of short distance ambulatory function (T25FW) is by far the most frequent functional loss (Figure 2). Isolated progression in cognition (as measured by the PASAT) or upper extremity function (as measured by the 9HP) was much less frequent. Importantly, the same pattern of progression over 2 years was observed regardless of whether the subjects had been clinically diagnosed as SPMS or PPMS (Figure 2). These findings may be helpful for the design of novel endpoints for clinical trials of progressive MS.

One limitation on the analysis of patient reported outcomes (PROs) is that information was available only for the subset of English speaking subjects from the IMPACT study (n = 127, 58%). However, the information available was useful to compare the rates of progression and improvement by subject self-report versus objective measurements in the context of SPMS. A comparison of domains represented both in objective measures (e.g. EDSS) and PROs (e.g. MSQLI) showed similar rates of improvement but in some cases higher rates of progression were elicited by subject’s report. For example, progression in bowel and bladder function was 13–16% by EDSS system (Table 3) versus 29–36% by MSQLI (Table 4). It is possible that some of the observed changes in the bowel and bladder system may be related to the use of symptomatic medication during the trials (e.g. anticholinergics, laxatives, etc; data not shown).

The findings of this analysis of longitudinal disease activity in the context of multicenter clinical trials support the view that PPMS and SPMS may be viewed as essentially similar. Although we used traditional definitions we also explored several novel definitions of disease activity. Further longer term studies are needed to validate their utility in clinical research and possibly in the clinic. It will also be important to confirm whether our findings are reproducible using different clinical trial data sets. For our analyses we were limited to study subjects with only 2 years of longitudinal follow up. It will also be important to examine clinical trial datasets with longer follow up. The analysis of disease activity at the individual subject level with a machine learning approach and heat map display shows great potential as a tool to study the natural progression and response to treatment of heterogeneous complex diseases like MS.
